# Pediatric Chronic Myeloid Leukemia Presenting in a Mixed Phenotypic Blast Crisis: A Rare Occurrence

**DOI:** 10.4274/tjh.galenos.2019.2018.0428

**Published:** 2019-08-02

**Authors:** Jenna Bhattacharya, Richa Gupta

**Affiliations:** 1 Maulana Azad Medical College, Department of Pathology, New Delhi, India

**Keywords:** Pediatric, Chronic myeloid leukemia, Blast crisis

## To the Editor,

**Figure 1:**
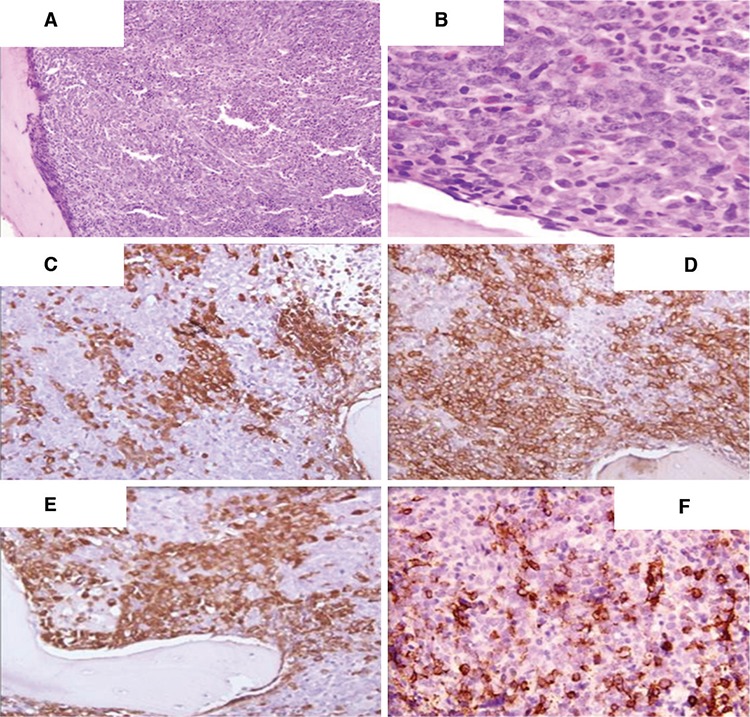
A) Bone marrow (BM) biopsy showing hypercellular marrow, H&E, 100^x^; B) BM biopsy showing blasts with prominent nucleoli, H&E, 400^x^; C) BM biopsy showing CD34 positivity in blasts, 400^x^; D) BM biopsy showing anti-MPO positivity in blasts, 400^x^; E) BM biopsy showing CD19 positivity in blasts, 400^x^; F) BM biopsy showing CD20 positivity in blasts, 400^x^.

Pediatric chronic myeloid leukemia (CML) comprises 3% of childhood leukemia cases [1]. Similar to adults, most of the patients present in the chronic phase, but 5% may present in a blast crisis (BC) [2]. Mixed phenotypic BC has rarely been reported in children [3]. A 10-year-old male presented with fever, fatigue, dull abdominal pain, and massive splenomegaly for 2 months. Complete blood count results were as follows: total leukocyte count (TLC), 544x109/L; hemoglobin, 8 g/dL; and platelet count, 80x109/L. Differential count on peripheral smear revealed the following: blasts - 25%, promyelocytes - 3%, myelocytes - 20%, metamyelocytes - 10%, eosinophils - 5%, basophils - 4%, monocytes - 2%, lymphocytes - 16%, and neutrophils - 15%. No dysplasia was noted. The blasts had moderate cytoplasm and prominent nucleoli. On cytochemistry, these blasts were negative for myeloperoxidase and periodic acid-Schiff. Bone marrow aspirate revealed a hypercellular marrow with myeloid predominance (M:E ratio of 25:1) with 55% blasts. Megakaryocytes were adequate with some dwarf forms. Bone marrow biopsy was hypercellular with near total replacement of marrow spaces with sheets of blasts having vesicular nuclei and prominent nucleoli (Figure 1). Blasts were positive for CD34, anti-MPO, CD19, and CD20 (Figure 1) and negative for CD3. Considering the high TLC and peripheral blood and bone marrow picture, RT-PCR for *M-BCR-ABL1* was done, which confirmed the presence of a 210-kDa transcript. Considering the clinical presentation, the peripheral blood picture (basophilia, many myelocytes and metamyelocytes), and the 210-kDa *BCR-ABL1* transcript, a diagnosis of mixed phenotypic BC in CML was issued and treatment was initiated with imatinib. Subsequently, the patient improved with lowering of TLC and disappearance of blasts from the peripheral blood. However, molecular response in follow-up could not be determined due to economic constraints.

The incidence of progression to BC in adults is 10% but the same is not well known in children [3]. BC is usually myeloid and rarely mixed phenotypic [4]. The biology of progression of pediatric CML to BC is supposed to be similar to that of adults [2]. Accumulation of additional chromosomal anomalies in the proliferating clone, especially deletions in the *CDKN2A/B *gene, deletions in the *IKZF* gene, and chromosomal aberrations associated with myelodysplasia, have been implicated with progression [5]. Mixed phenotypic BC in CML needs to be differentiated from de novo mixed phenotypic acute leukemia (MPAL). Differentiation may be difficult since MPAL can also show *M-BCR-ABL1* translocation [6]. The points in favor of CML include high TLC at presentation, massive splenomegaly, peripheral blood basophilia and all myeloid precursors, absence of dysplasia, and the 210-kDa transcript on PCR. In such cases, if *M-BCR-ABL1* fusion signals are detected by FISH/PCR in mature neutrophils as well as in blasts (present in our case), then CML-BC is the most likely diagnosis [3]. Moreover, in a case of MPAL, if post-induction RT-PCR shows a high number of abl-bcr transcripts despite reduction in blast count, the diagnosis of BC in CML should be considered. The progression to blast phase warrants a poor prognosis in CML, which is further worsened by the presence of the mixed phenotypic type of BC [3]. Such cases should be treated with tyrosine kinase inhibitors plus chemotherapy based on the blast phenotype [4,7].
